# Selenide-Containing Polyimides with an Ultrahigh Intrinsic Refractive Index

**DOI:** 10.3390/polym10040417

**Published:** 2018-04-09

**Authors:** Qilong Li, Jiandong Zhang, Xiangqiang Pan, Zhengbiao Zhang, Jian Zhu, Xiulin Zhu

**Affiliations:** 1 State and Local Joint Engineering Laboratory for Novel Functional Polymeric Materials, College of Chemistry, Chemical Engineering and Materials Science, Soochow University, Suzhou 215123, China; qlli@stu.suda.edu.cn (Q.L.); zhangjiandong@suda.edu.cn (J.Z.); xlzhu@suda.edu.cn (X.Z.); 2 Jiangsu Key Laboratory of Advanced Functional Polymer Design and Application, College of Chemistry, Chemical Engineering and Materials Science, Soochow University, Suzhou 215123, China

**Keywords:** polyimide, selenium element, high refractive index, low reflection, polycondensation

## Abstract

This work developed novel selenium-containing polyimides with a high intrinsic refractive index. Four polyimides with different selenium contents and repeat unit structures were designed and synthesized via amine-dianhydride polycondensation of one of two diamines, i.e., 4,4′-oxydianiline or *bis*(4-aminophenyl)selanide, with one of two dianhydrides, i.e., *bis*(4-(3,4-dicarboxylbenzoyloxy)phenyl) ester dianhydride or 1,1′-*bis*(4-(3,4-dicarboxylbenzoyloxy)phenyl) selenide dianhydride. Various techniques, e.g., nuclear magnetic resonance, Fourier transformed infrared spectroscopy, and wide-angle X-ray diffraction, were used to characterize the polymers’ structures. Differential scanning calorimetry, thermogravimetric analysis, ultraviolet-visible spectroscopy, and spectroscopic ellipsometry were used to characterize the properties of the polymers. The selenium contents showed a positive effect on the refractive index of the final polymer. In addition, the refractive index can reach up to 1.968 at 633 nm, which was the highest intrinsic refractive index of a polyimide ever reported. Because of the high intrinsic refractive index, the reflective ratio of visible light on the surface of a silicon wafer was significantly reduced, indicating the potentially utility of the polymer in an anti-reflection coating.

## 1. Introduction

Synthetic polymers with a high refractive index (RI) are very useful in optics applications, such as advanced display devices, various lenses, optical waveguides, and diffractive gratings [1,2,3,4,5,6]. Based on the Lorentz-Lorenz equation [7], polymeric materials with a high RI can be developed by introducing moieties with a high molar refraction and a low molar volume, such as aromatic rings, halogens, and phosphorus- and sulfur-containing groups [8,9,10,11]. Minns et al. developed novel polymers with a high RI (1.670–1.770) through the incorporation of halogens into the polymer structures [12]. The results indicated that the RI increases with increasing halogen content. However, halogen-containing polymers were recognized as unfriendly to the environment, limiting their potential applications. Due to the high polarizability of phosphorus, phosphorus-containing polymers such as polyphosphazenes and polyphosphonates show high RI values. Allcock et al. reported the high RI of some phosphorus-containing materials with different phosphorus side groups, e.g., a polyphosphazene with an RI of 1.664–1.755, and a polyphosphonate with an RI of 1.600–1.639 [13,14,15,16]. Recently, Tu et al. reported a novel method for fabricating high RI polymers (*n* > 1.80) by incorporating fullerene moieties [17,18]. Among the various polymers with high RI values, sulfur-containing polyimides (PIs) have received much attention due to their excellent thermal stability, low dielectric constant, and outstanding mechanical properties [19,20]. RI values greater than 1.70 have been demonstrated for sulfur-containing PIs with different groups [21,22,23,24,25]. Recently, Ueda et al. thoroughly summarized the literature on sulfur-containing PIs. High sulfur and aromatic contents along with low molecular volumes in sulfur-containing PIs resulted in not only high RI but also low birefringence, which makes them more appealing for various potential applications [26]. Selenium, an analog of sulfur in the chalcogen group, possesses much higher molar refraction ([R]) even than sulfur, i.e., [R] = 11.17 cm^3^/mol of selenide and [R] = 7.69 cm^3^/mol of sulfur [27]. Ueda et al. conducted excellent work on selenophene-containing PIs with a high RI of 1.759 [28]. Recently, Pyun’s group reported the synthesis of selenium-containing materials showing ultrahigh RI (>2.0) in the IR range [29,30]. These inorganic/organic polymeric hybrid materials contained up to 42 wt % of gray selenium. However, regarding the RI values within the visible range, available data were scarce. The obtained material can be potentially applied to imaging with a mid-IR camera under ambient conditions [31]. Our group also developed different kinds of selenium-containing polymers in recent years [32,33]. By introducing selenium-containing moieties into the polymer chain, both selenium-containing polystyrene and poly(methyl acrylate)-based polymers were synthesized with controlled structures and RI values greater than 1.70. Furthermore, the RI of the obtained polymers could be adjusted using the combination of controlled radical polymerization techniques and post-modification, giving an RI range of 1.611–1.719. These results demonstrated that selenium in polymer can enhance its RI value. High RI PI materials combine the advantages of high RI polymers with those of PIs, such as their light weight and improved stability compared to inorganic/organic polymeric hybrid materials. Thus, it would be valuable to further improve the RI of PIs. Little work has been done on polymers with selenide incorporated into the backbone as high RI materials, especially in the development of polymers containing organoselenium moieties for the fabrication of intrinsic high RI polymers. 

Herein, we report four novel PIs with selenide-containing moieties in the backbone to obtain high RI materials. The syntheses of the four monomers were performed according to the literature [34,35], and the detailed synthetic procedures with optimized conditions are shown in the Supporting Information, as well as the novel selenide-containing diamine and dianhydride monomers (Scheme S1). All compounds were accurately characterized with nuclear magnetic resonance (NMR), high resolution mass spectrum (HRMS), and elemental analysis (EA) and are summarized in the Supporting Information.

## 2. Materials and Methods 

### 2.1. Materials

4-Iodoaniline (98%), CuI (98%), 4,4′-oxydiphenol (ODP, 98%), and 4,4′-oxydianiline (ODA, 99%) were purchased from Energy Chemical, Shanghai, China. Ethyl acetate (AR), NaBH_4_ (98%), chloroform (AR), and acetone (AR) were purchased from Yonghua (Jiangsu) Technology Co., Ltd., Changshu, China. Se powder (99%) was purchased from Shanghai Meixing Chemical, Shanghai, China. Sulfuryl chloride (AR) was purchased from Shanghai Jinshan Ting Xin Chemical Co., Ltd., Shanghai, China. Phenol (AR), *N*,*N*-dimethylacetamide (DMAc, AR), *N*-methyl pyrrolidone (NMP, AR), *m*-cresol (AR), and acetic anhydride (Ac_2_O, AR) were purchased from Sinopharm Group Chemical Reagent Co., Ltd., Ningbo, China. Anhydrous K_2_CO_3_ (AR), pyridine (AR), dimethylsulfoxide (DMSO) (AR), anhydrous Na_2_SO_4_ (AR), toluene (AR), anhydrous ethyl ether (AR), and Na_2_CO_3_ (AR) were purchased from Chinasun Specialty Products Co., Ltd., Changshu, China. Trimellitic anhydride chloride (TMAC, 98%) was purchased from Tokyo Chemical Industry, Tokyo, Japan. All of the chemicals mentioned above were used as received without further purification. Tetrahydrofuran (THF), purchased from Sinopharm Group Chemical Reagent Co., Ltd., Ningbo, China, was distilled from calcium hydride under nitrogen immediately before use. Pyridine and *N*,*N*-dimethylformamide (DMF), purchased from Sinopharm Group Chemical Reagent Co., Ltd., Ningbo, China, were dried by molecular sieve. Textured silicon wafers were supplied by the College of Physics, Optoelectronics, and Energy of Soochow University, Suzhou, China. 

### 2.2. Measurement

^1^H-NMR and ^13^C-NMR spectra were measured on a Bruker 300 MHz spectrometer using DMSO-*d*_6_ as the solvent and tetramethylsilane (TMS) as an internal standard. ^77^Se-NMR spectra were measured on Agilent Technologies 600 MHz spectrometer (Santa Clara, CA, USA) using DMSO-*d*_6_ as the solvent and tetramethylsilane (TMS) as an internal standard. Elemental analysis was performed on a Perkin-Elmer-240 (II) C, H, N elemental analyzer (Waltham, MA, USA). Mass Spectrometry (MS) was measured on Bruker microTOF-QIII spectrometer (Karlsruhe, Germany), using acetonitrile as the solvent. Thermogravimetric analysis (TGA) measurements were conducted with a Perkin-Elmer TGA-2 (Waltham, MA, USA) in flowing nitrogen (20 mL/min) at a heating rate of 10 °C/min. The differential scanning calorimetry (DSC) measurements were conducted with a TA Instruments DSC Q20 (New Castle, DE, USA) in flowing nitrogen (50 mL/min) at a heating rate of 10 °C/min. Wide angle X-ray diffraction (WAXD) was carried out with an PANalytical X’Pert-Pro MPD X-ray diffractometer (Egham, Surrey, UK). Fourier transform infrared spectroscopy (FT-IR) of film samples were recorded with the Bruker TENSOR 27 FT-IR Instrument (Karlsruhe, Germany). Ultraviolet visible (UV-Vis) spectra of the film samples were recorded on a Shimadzu UV-2600 spectrophotometer (Kyoto, Japan). The highest occupied molecular orbital (HOMO) and lowest unoccupied molecular orbital (LUMO) of repeat units in these four PIs were calculated using the three parameter hybrid B3LYP density functional method with the extended basis set 6-311++G(d,p) implemented in the Gaussian 08 package. Scanning electron microscope patterns were measured by a Hitachi SU5000 SEM (Tokyo, Japan). The refractive index and thickness of PI films were measured by M2000, J.A. Woollam Co. (Lincoln, NE, USA) spectroscopic ellipsometer with 70-degree angle of incidence. The in-plane (*n*_TE_) and out-of-plane (*n*_TM_) refractive index of the PI films were measured. The mean refractive index (*n*_av_) was calculated using the equation *n*_av_^2^ = (2*n*_TE_^2^ + *n*_TM_^2^)/3. The in-plane/out-of-plane birefringence (Δ*n*) was calculated by using the equation Δ*n* = *n*_TE_ − *n*_TM_. Reflectivity was measured by Shanghai Ideaoptics (China) R9000 Integral Reflectometer (Shanghai, China). The thin PI films were prepared as follows: monocrystalline/textured silicon wafers were subjected to vacuum adsorption on a rotating plate of a KW-4A type Spin Coater (Shanghai, China), and then polyamide acid (PAA) samples were dropped on the monocrystalline/textured silicon wafers, following which the spin coater was employed at a rate of 3000 r/min, held for 30 s, and finally the product was thermally imidized.

### 2.3. Monomer Synthesis

The detailed synthetic procedure of the four monomers was performed as follows (Scheme S1):

Synthesis of *bis*(4-aminophenyl)selenide (BAPSe): BAPSe was synthesized according to the literature with optimized conditions [36,37]. A 50-mL three-necked flask containing a magnetic stirring bar was charged with 4-iodoaniline (2.19 g, 10 mmol), CuI (0.19 g, 1 mmol), K_2_CO_3_ (1.38 g, 10 mmol), DMSO (20 mL), and Se powder (0.47 g, 6 mmol) under an argon flow. The mixture was heated for 12 h at 120 °C and allowed to cool to room temperature. After filtration, the resulting mixture was extracted with ethyl acetate (3 × 25 mL). The combined organic layers were dried with anhydrous Na_2_SO_4_ and then concentrated under vacuum. The residue was purified by column chromatography on silica gel with an eluent consisting of petroleum ether and ethyl acetate (petroleum ether:ethyl acetate = 4:1 (*v*/*v*)) to give the product as a brown solid (1.02 g, 39.5%). ^1^H-NMR (300 MHz, DMSO-*d*_6_, δ): 7.09 (d, *J* = 8.0 Hz, 4H), 6.47 (d, *J* = 8.0 Hz, 4H), 5.18 (s, 4H) (Figure S1). ^13^C-NMR (75 MHz, DMSO-*d*_6_, δ): 148.83, 134.70, 116.15, 115.20 (Figure S2). ^77^Se-NMR (114 MHz, DMSO-*d*_6_): 370.49 (Figure S3). HRMS (ES+) calcd for C_12_H_12_N_2_Se [M + H]^+^: 265.0166, found: 265.0236. Anal. Calcd for C_12_H_12_N_2_Se: C, 55.54; N, 10.61; H, 4.59. Found: C, 55.16; N, 10.55; H, 4.50.

Synthesis of *bis*(4-hydroxyphenyl)selenide (BHPSe): To a mixture of Se powder (0.79 g, 10 mmol) and chloroform (20 mL) was added a solution of sulfuryl chloride (1.35 g, 10 mmol) in 20 mL of chloroform. After the complete dissolution of selenium power, a mixture of phenol (1.88 g, 20 mmol) and anhydrous ethyl ether (20 mL) was added. Then the mixture was stirred at 25 °C for 3 h. Then a solution of 1.06 g of Na_2_CO_3_ in 4 mL water was added dropwise. The organic layer was separated and subsequently washed with H_2_O (3 × 15 mL). The organic layer was dried with anhydrous Na_2_SO_4_, and the solvent was removed under reduced pressure to give a yellow solid. The crude product was purified by column chromatography on silica gel with an eluent consisting of petroleum ether and ethyl acetate (petroleum ether:ethyl acetate = 8:1 (*v*/*v*)) giving BHPSe as a yellow solid (1.52 g, 56.9%) ^1^H-NMR (300 MHz, DMSO-*d*_6_): 9.58 (s, 2H), 7.24 (q, 4H), 6.70 (q, 4H) (Figure S4). ^13^C-NMR (75 MHz, DMSO-*d*_6_): 157.65, 134.99, 119.76, 117.01 (Figure S5). ^77^Se-NMR (114 MHz, DMSO-*d*_6_): 377.9 (Figure S6). HRMS (ES+) calcd for C_12_H_10_O_2_Se [M + H]^+^: 264.9846, found: 264.8935. Anal. Calcd for C_12_H_10_O_2_Se: C, 55.180; N, 0.000; H, 3.793. Found: C, 54.350; N, 0.000; H, 3.809.

Synthesis of 1,1′-*bis*(4-(3,4-dicarboxylbenzoyloxy)phenyl) selenide dianhydride (BDPSD) and 1,1′-*bis*(4-(3,4-dicarboxylbenzoyloxy)phenyl) ester dianhydride (BDPED): BHPSe (2.65 g, 10 mmol) was dissolved in dry THF (20 mL) and pyridine (2.37 g, 30 mmol) in a 100-mL three-necked flask. TMAC (0.42 g, 20 mmol) was dissolved in 30 mL dry THF and added dropwise to the solution at 0 °C. After stirring at room temperature for 24 h, the mixture was separated by filtration. The filtrate was concentrated with an evaporator and slowly poured into water. The precipitate was obtained after filtration and vacuum-dried at 80 °C for 12 h. The partially hydrolyzed product was added to an excess of Ac_2_O and heated for 3 h at 120 °C to ensure cyclodehydration. The crude product was recrystallized from toluene and Ac_2_O (*v*/*v* = 6:1), giving BDPSD. ^1^H-NMR (300 MHz, DMSO-*d*_6_): 8.61 (m, 4H), 8.27 (d, *J* = 7.9 Hz, 2H), 7.62 (m, 4H), 7.40 (m, 4H) (Figure S7). ^77^Se-NMR (114 MHz, DMSO-*d*_6_): 404.93 (Figure S8). Anal. Calcd for C_30_H_14_O_10_Se: C, 58.740; N, 0.000; H, 2.300. Found: C, 57.770; N, 0.000; H, 2.527. IR (KBr): 1849, 1786 (anhydride C=O), 1743 (ester C=O stretching), 1230 (C–O–C) cm^−1^. BDPED was obtained from the same procedure, using 4,4′-Oxydiphenol (ODP) instead of BHPSe. ^1^H-NMR (300 MHz, DMSO-*d*_6_): 8.62 (m, *J* = 10.9, 3.0 Hz, 4H), 8.28 (d, *J* = 8.6 Hz, 2H), 7.44 (m, *J* = 16.9, 9.0 Hz 4H), 7.17 (m, 4H) (Figure S9). Anal. Calcd for C_30_H_14_O_11_: C, 66.750; N, 0.000; H, 2.730. Found: C, 65.450; N, 0.000; H, 2.564. IR (KBr): 1861, 1784 (anhydride C=O), 1731 (ester C=O stretching), 1227 (C–O–C) cm^−1^.

### 2.4. Preparation of the Polyimide Films

The polyimide films were prepared by conventional two-step polycondensation followed with thermal imidization (Scheme S2). The general synthetic process for preparing the polyimide is summarized as follows. BDPSD (1.226 g, 2 mmol) was added to the solution of BAPSe (0.526 g, 2 mmol) in DMF (11 mL). After stirring at room temperature for 3 h, the solution was poured onto a glass plate and dried at 100 °C for 1 h, 150 °C for 1 h, 200 °C for 1 h, 250 °C for 2 h, and 300 °C for 0.5 h. After being soaked in boiled water, a flexible polyimide film with a thickness of around 30 μm was released from the glass surface.

## 3. Results and Discussion

**Figure 1:**
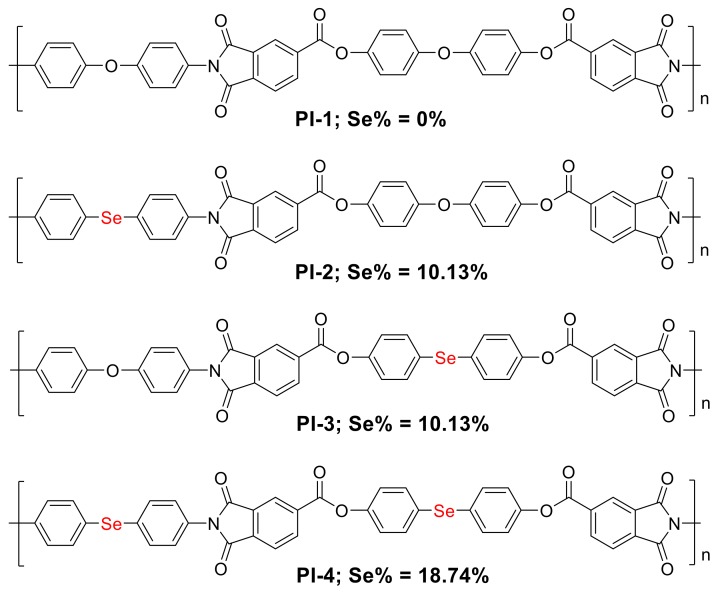
The structures of four polyimides (PIs) with different contents structures; Se% indicates the theoretical mass contents.

Through the rational design of the monomer structures, PIs with different selenide contents and structures were synthesized as shown in Figure 1. PI films with a thickness of approximately 30 μm were prepared by conventional two-step polycondensation followed by thermal imidization (Scheme S2). The structures of these polyamide acids (PAAs) and PIs were characterized by Fourier transform infrared (FT-IR) spectroscopy (Figure S10). PAA samples showed a characteristic absorption band at 3450 cm^−1^, which is attributed to the strong intramolecular modes of the hydroxyl moiety in the carboxyl groups. The absorption bands at 1660, 1530, and 1250 cm^−1^ correspond to −CO−NH− stretching. After thermal imidization, the characteristic bands of the carboxyl groups in the PAAs at 3450 cm^−1^ disappeared, which indicated a complete imidization reaction after the heat treatment. In addition, new absorption bands at 1720, 1370, and 740 cm^−1^ corresponding to imide stretching were observed, demonstrating the formation of the PIs.

**Figure 2:**
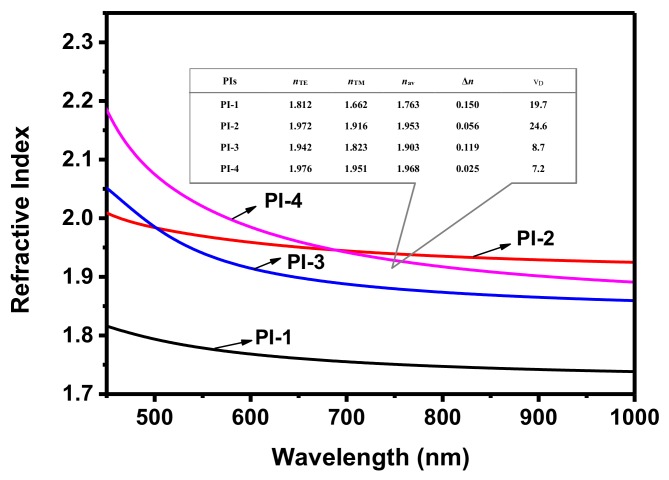
Average refractive index curves of four synthesized polyimides at different wavelengths. (*n*_TE_, in-plane refractive indices and *n*_TM_, out-of-plane refractive indices measured at 633 nm; *n*_av_, average refractive indices calculated using the equation *n*_av_^2^ = (2*n*_TE_^2^ + *n*_TM_^2^)/3. Δ*n*, the in-plane/out-of-plane birefringence calculated using the equation Δ*n* = *n*_TE_ − *n*_TM_. v_D_, average Abbe number calculated by the equation v*_D_* = (*n*_D_ − 1)/(*n*_F_ − *n*_C_), *n*_D_, *n*_F_, and n_C_ represented average refractive indices at 589 nm, 486 nm, and 656 nm, respectively.).

The RIs of the as-prepared PI films were measured by spectroscopic ellipsometry, and the results are summarized in Figure 2. The RI of PI-1 without selenide incorporation was 1.763 at a wavelength of 633 nm. After the introduction of selenium, the RI increased dramatically to 1.953, 1.903, and 1.968 for PI-2, PI-3, and PI-4, respectively. These results clearly indicated that the introduction of selenium moieties can efficiently improve the RI of PIs, which was in agreement with the literature [32,33]. The position of selenide in the polymer backbone also showed interesting effects on the RI value. A higher RI could be obtained when selenium was located in the diamine (PI-2, *n* = 1.953) than when it was located in the dianhydride (PI-3, *n* = 1.903), which may be due to the higher deformability of the diamine than the dianhydride in PIs. PI-4 with selenium in both the diamine and dianhydride repeating units showed the highest RI, i.e., *n* = 1.968, which was the highest intrinsic RI of a polymer ever reported.

The Abbe number is another important optical parameter for optical materials. Typically, a high-polarity molecule will present a high wavelength dispersion and a low Abbe number. The Abbe numbers for PI-1, PI-2, PI-3, and PI-4 are 19.7, 24.6, 8.7, and 7.2, respectively. In general, higher selenium content resulted in a lower Abbe number. However, among these four PIs, PI-2 exhibited the highest Abbe number of 24.6. These results implied that introducing selenium into the diamine repeating unit is an effective means of obtaining polymers with both a high Abbe number and a high RI. The PIs were deep yellow due to the existence of a charge-transfer (CT) structure formed by the diamine donor moieties and dianhydride acceptor moieties [36,37]. To investigate the optical properties, the four PIs were characterized by UV-Vis spectrometry (Figure S11). Clearly, the transmittance for all four samples was almost 100% when the wavelength was greater than 500 nm, and close to zero when the wavelength was less than 300 nm. The main differences in the UV-Vis spectra of these four synthesized PIs were in the wavelength range from 300 to 500 nm. With increasing selenium content, the transmittance at shorter wavelengths decreased rapidly. For example, the transmittance (%) at 400 nm of PI-1, PI-2, PI-3, and PI-4 was 45.2, 19.5, 32.6, and 0.9, respectively. To obtain a better understanding, a computer simulation was carried out by using the three-parameter hybrid B3LYP density functional method with the extended basis set 6-311++G(d,p) implemented in the Gaussian 08 package (Tables S1 and S2). [38] Both the increasing trend in the absorption edges (*λ_E_*) and the decreasing trend in the energy band gaps (*Δε*) follow the order PI-1, PI-3, PI-2, and PI-4, which matches the above results. Although the cut-off wavelength was increased a little after the introduction of selenium moieties into the PI repeating units, all of the PI films appeared almost transparent upon irradiation at *>*500 nm, which would be useful for the fabrication of optical devices. The solubility of these four PIs in different common organic solvents was investigated (Table S3). The results showed that all four PIs are insoluble in common organic solvents at room temperature and when heated. Many factors, such as the flexibility of polymer chains and structure and arrangement of polymer repeating units, influence polymer solubility. To investigate their aggregation structure, these PI films were characterized by wide-angle X-ray diffraction (WAXD) (Figure S12). The 2θ of the major peak is centered at 18.71°, 18.61°, 19.63°, and 19.76° for PI-1, PI-2, PI-3, and PI-4, respectively, indicating that the packing distance increased from PI-1 to PI-4. Thus, after the introduction of selenium into the PIs, the stacking intensity was decreased. The peak intensity decreased from PI-1 to PI-4, which indicated that the crystallinity of these polymers decreased from PI-1 to PI-4. This result showed that the dihedral angle and linear length of the repeating unit modeled in the HOMO state were different after the introduction of selenium, which led to obvious torsion of the skeleton of the selenium-containing PIs. This change made it difficult for the polymer chains to tightly pack, which resulted in the observed decrease in crystallinity [39]. The thermal properties of these PIs were characterized by thermogravimetric analysis (TGA) and differential scanning calorimetry (DSC) (Table S4). Detailed curves are shown in Figures S13 and S14. Normally, polypyromellitimides show excellent thermal properties, including decomposition temperatures up to 400 °C and glass transition temperatures up to 250 °C [40,41,42,43]. Excellent thermal properties for polypyromellitimides have been recognized to result from the characteristic architecture of these polymers, i.e., strong rigidity of the main chain and high rigidity of the repeating units. From PI-1 to PI-4, the *T*_d_^5^ (5% decomposition temperature) was 417, 411, 376, and 356 °C, and the *T*_d_^10^ (10% decomposition temperature) was 457, 456, 438, and 438 °C, respectively. These results indicated that the decomposition temperatures of the polymers decreased as the selenium content, especial the value of *T*_d_^5^, which may be attributable to the thermal instability and low bond angle of selenium. PI-2 and PI-3 with a single selenium atom in different places in the repeating unit have lower decomposition temperatures than selenium-free PI-1. In particular, PI-4 with the selenium contents showed the lowest decomposition temperature (356 °C) among these four PIs. Regarding the glass transition temperature (*T*g), the results showed a similar trend with increasing PI selenium content, i.e., 267, 238, 258, and 205 °C for PI-1, PI-2, PI-3, and PI-4, respectively. After one of the oxygen atoms in PI-1 was replaced by selenium in PI-2, the *T*g value rapidly decreased from 267 to 238 °C. Furthermore, the position of the selenide also showed an interesting effect on the *T*g of the polymers. PI-2 with the selenide in the diamine structure showed a higher *T*g than PI-3 with the selenide in the dianhydride structure. PI-4 with the maximum selenium content had the lowest *T*g value of 205 °C, because selenium has a larger element volume and a smaller bond angle than oxygen, resulting in higher flexibility. However, the *T*g of the four PIs were higher than 200 °C, demonstrating their good thermal properties, which are favorable for their potential applications.

**Figure 3:**
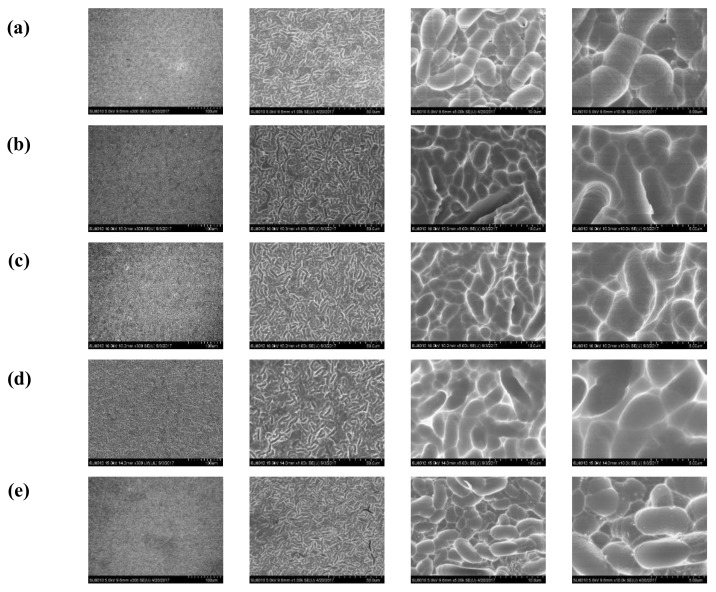
SEM images of a textured silicon wafer (**a**) and four polyimides (PIs) spin-coated on textured silicon wafers ((**b**) PI-1; (**c**) PI-2; (**d**) PI-3; (**e**) PI-4) under different enlargement ratios.

**Figure 4:**
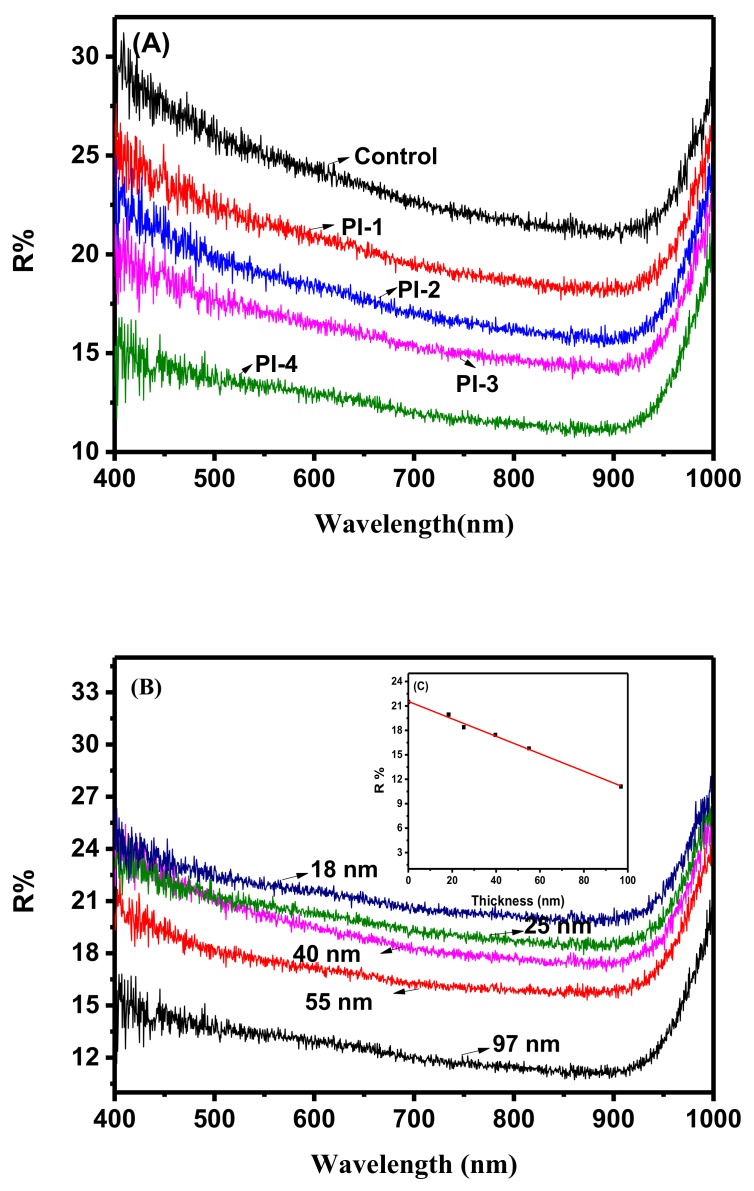
Reflectivity of textured silicon wafers with and without (as a control) the cover of a PI film under different conditions: (**A**) different PIs with a film thickness of 100 ± 5 nm; (**B**,**C**) relationships between R% and PI4 film thickness.

An important application of high RI materials is the fabrication of anti-reflection coating (ARC). In this study, to verify the ARC effect of these four PIs, the PAA solutions were spun onto textured silicon wafers followed by thermal imidization. Thus, PI films were coated onto the surface of the textured silicon wafer. Repeating this process allowed the thickness of the film to be adjusted. The surface morphology of the silicon wafer before and after spin-coating was characterized by SEM. The results, which are summarized in Figure 3, show that the surface morphology was similar before and after the spin-coating process, which provided the ideal samples for investigating the effect of RI on reflectivity. The surface reflectivity of the samples covered with different PIs with a similar film thickness of approximately 100 nm to light with wavelengths ranging from 400 to 1000 nm was characterized. The results are shown in Figure 4A. Obviously, the reflectivity of the wafers to the light with wavelengths ranging from 400 to 1000 nm was dramatically decreased after they were covered by PI films. Increasing the RI of the PI will decrease the reflectivity of the surface. Reflectivity as low as approximately 11% at a wavelength of 850 nm was achieved after the silicon wafer was covered with a PI-4 film. Simultaneously, the effect of film thickness on its reflectivity was investigated, and the results are shown in Figure 4B. The reflectivity gradually decreased with increasing film thickness, e.g., from 21.48% for the original wafer to 19.93%, 18.38%, 17.45%, 15.79%, and 11.15% when the thickness increased to 18.4 nm, 25.3 nm, 39.7 nm, 55.0 nm, and 96.9 nm, respectively. The effect of thickness on the reflectivity was confirmed to be a linear relationship, as shown in Figure 4C, indicating the regulation of film thickness as an alternative and facile way for adjusting reflectivity.

## 4. Conclusions

In this work, novel selenium-containing PI films were designed and synthesized. The four PIs showed high RI values in the range of 1.763–1.968 at 633 nm. Increasing selenium content resulted an increase in RI. In particular, PI-4 with the highest selenium content had the highest RI of 1.968, which is the highest value ever reported for a polymeric material. These PIs with a high RI could be used as AR coatings showing almost no change in surface morphology and providing a low reflectivity value of approximately 11% at 850 nm.

## Supplementary Materials

The following are available online at http://www.mdpi.com/2073-4360/10/4/417/s1, Figure S1: ^1^H-NMR of BAPSe in DMSO-*d*_6_. Figure S2: ^13^C-NMR of BAPSe in DMSO-*d*_6_. Figure S3. ^77^Se-NMR of BAPSe in DMSO-*d*_6_. Figure S4. ^1^H-NMR of BHPSe in DMSO-*d*_6_. Figure S5. ^13^C-NMR of BHPSe in DMSO-*d*_6_. Figure S6. ^77^Se-NMR of BHPSe in DMSO-*d*_6._ Figure S7. ^1^H-NMR of BDPSD in DMSO-*d*_6_. Figure S8. ^77^Se-NMR of BDPSD in DMSO-*d*_6_. Figure S9. ^1^H-NMR of BDPED in DMSO-*d*_6_. Figure S10. FT-IR spectra of four synthesized PAA (PAA1-4) (A) and four synthesized PI (PI1-4) (B) films on a potassium bromide tablet. Figure S11. UV-Vis spectra of four synthesized PIs with about a 2-μm thickness. Figure S12. WAXD profiles of four synthesized PIs. Figure S13. TGA thermographs of four synthesized PIs with N2 atmosphere. Figure S14. DSC thermographs of four synthesized PIs with N2 atmosphere. The glass transition temperatures of the four PIs are labeled. Scheme S1. Synthetic route of monomers (a, synthesis route of BAPS; b, synthesis route of BHPS, BDPSD, and BDPED). Scheme S2. Preparation procedures and the structures of four PIs with different contents and locations of selenium in their repeat units. Table S1. Optical transmittance, absorption edges (λ_E_), calculated molecular orbital energies (ε) of HOMO and LUMO and energy band gaps (Δε) for monomeric models of four synthesized PIs. Table S2. Molecular orbital (MO) diagrams of four synthesized PIs. Table S3. Solubility of four synthesized PIs. Table S4. Thermal properties of four synthesized PIs.
