# Supplementary material for: Selenide-Containing Polyimides with an Ultrahigh Intrinsic Refractive Index
Source: Polymers (Basel). 2018 Apr 9;10(4):417. doi: 10.3390/polym10040417 (PMC6415271; doi:10.3390/polym10040417)
Supplement: Supplementary file 1 [file polymers-10-00417-s001.pdf]

Supporting Information

Selenium-containing Polyimides with an Ultrahigh Intrinsic Refractive Index

Qilong Li, Jiandong Zhang, Dr. Xiangqiang Pan*, Prof. Dr. Zhengbiao Zhang*, Prof. Dr. Jian Zhu*, Prof. Dr. Xiulin Zhu

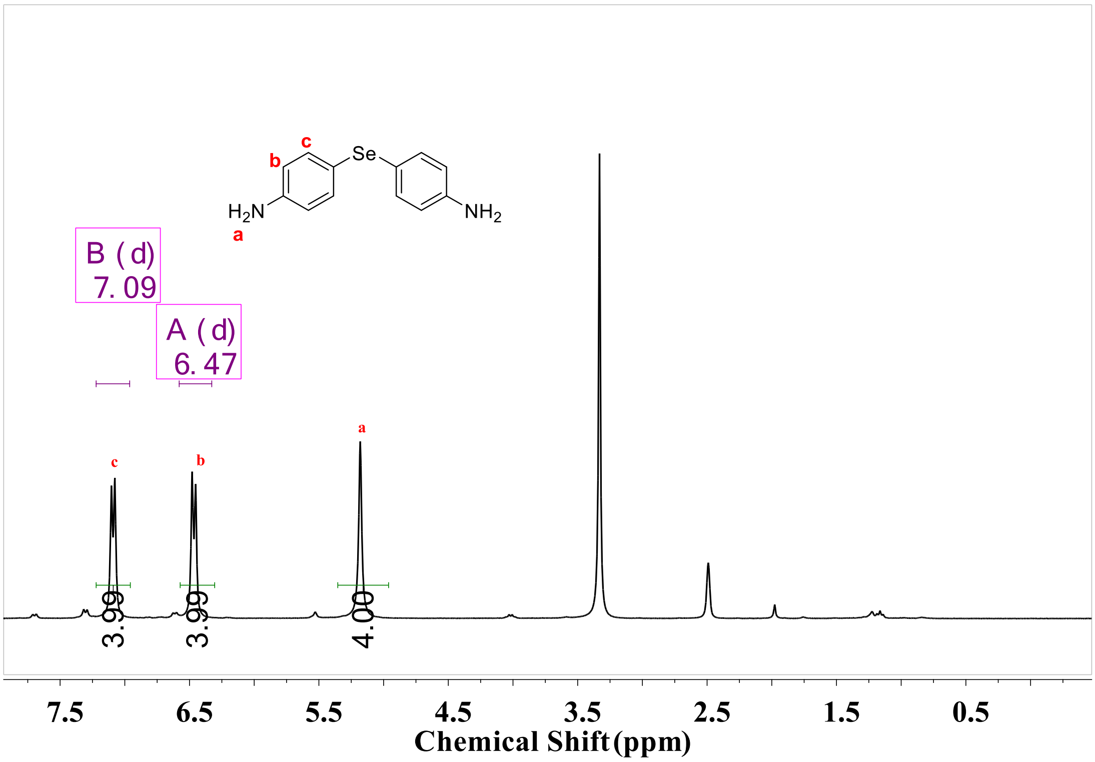

Figure S1. ^{1}H NMR of BAPSe in DMSO-d_6.

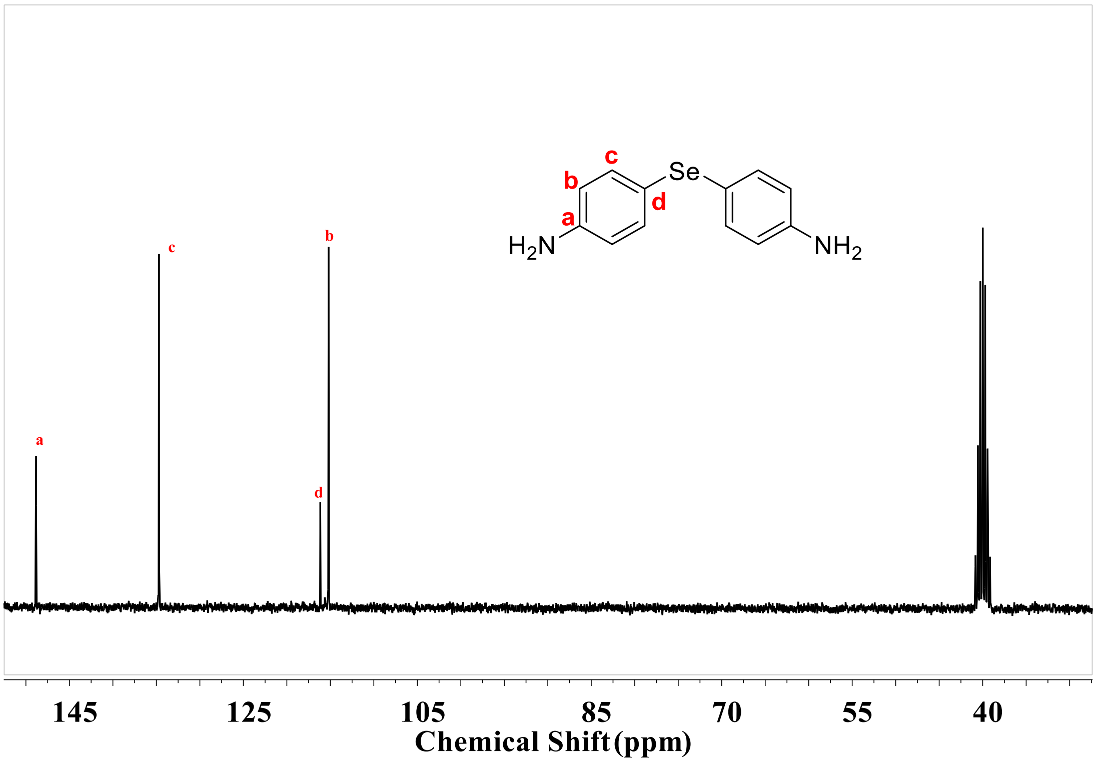

Figure S2. ^{13}C NMR of BAPSe in DMSO-d_6.

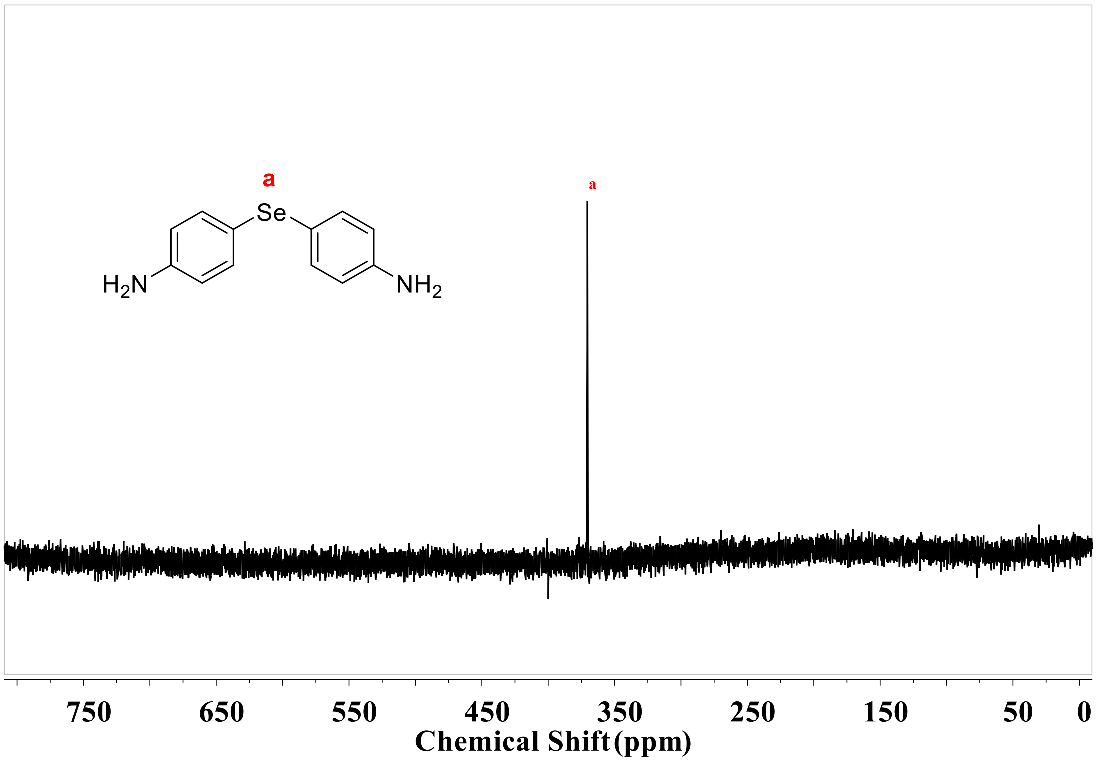

Figure S3. ^{77}Se NMR of BAPSe in DMSO-d$_6$.

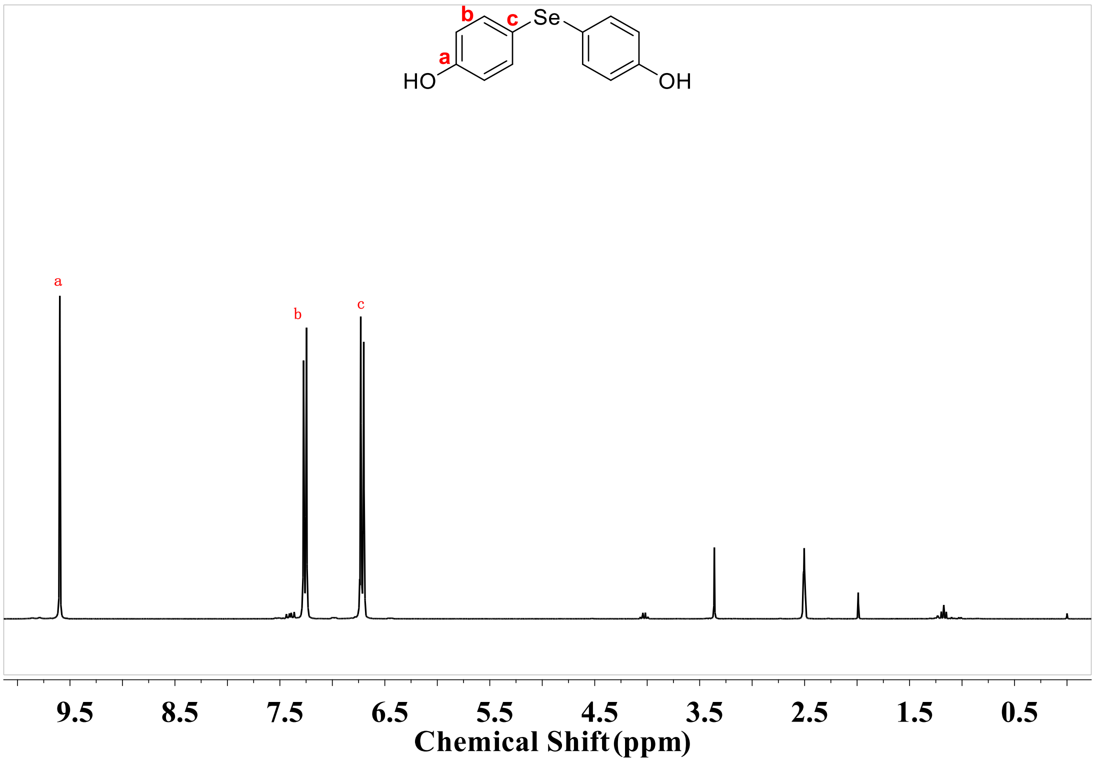

Figure S4. ^{1}H NMR of BHPSe in DMSO-d$_6$.

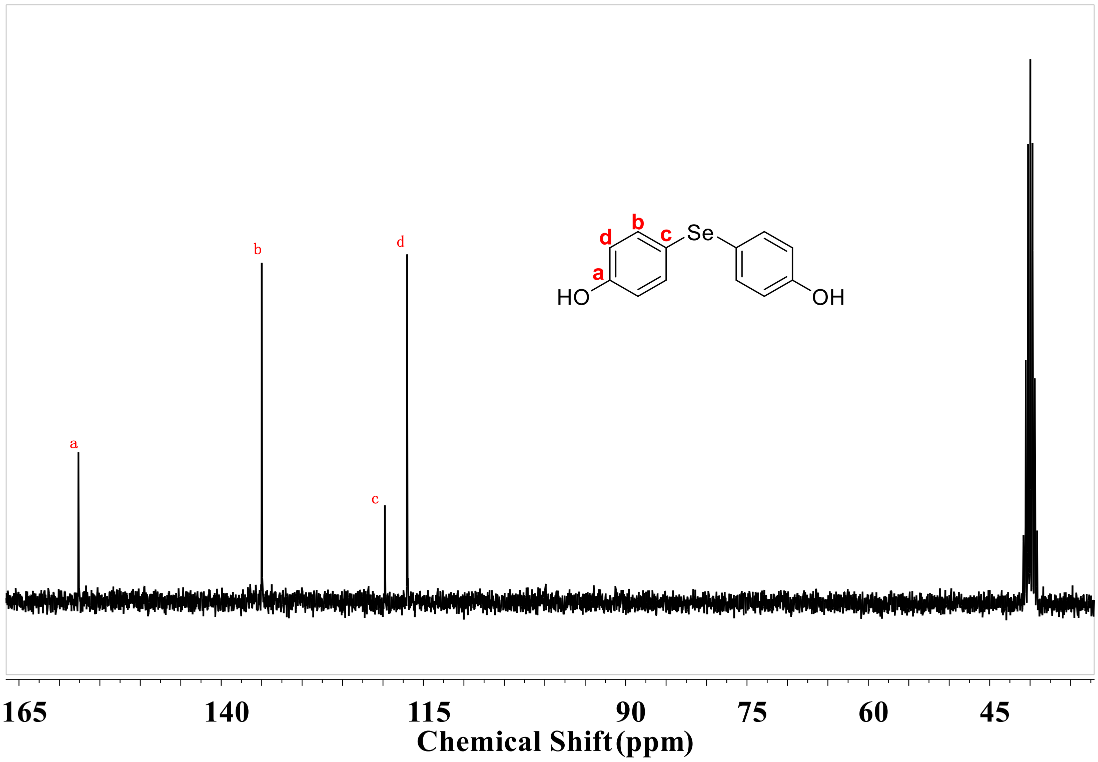

Figure S5. ^{13}C NMR of BHPSe in DMSO-d_6.

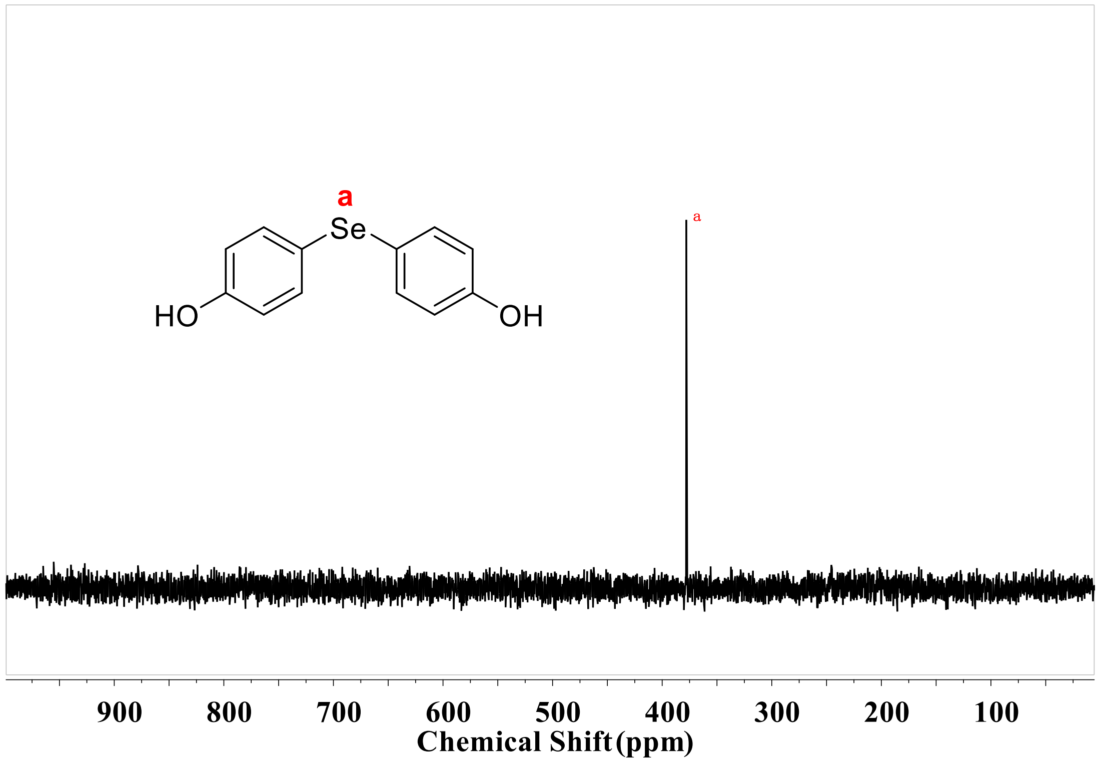

Figure S6. ^{77}Se NMR of BHPSe in DMSO-d_6.

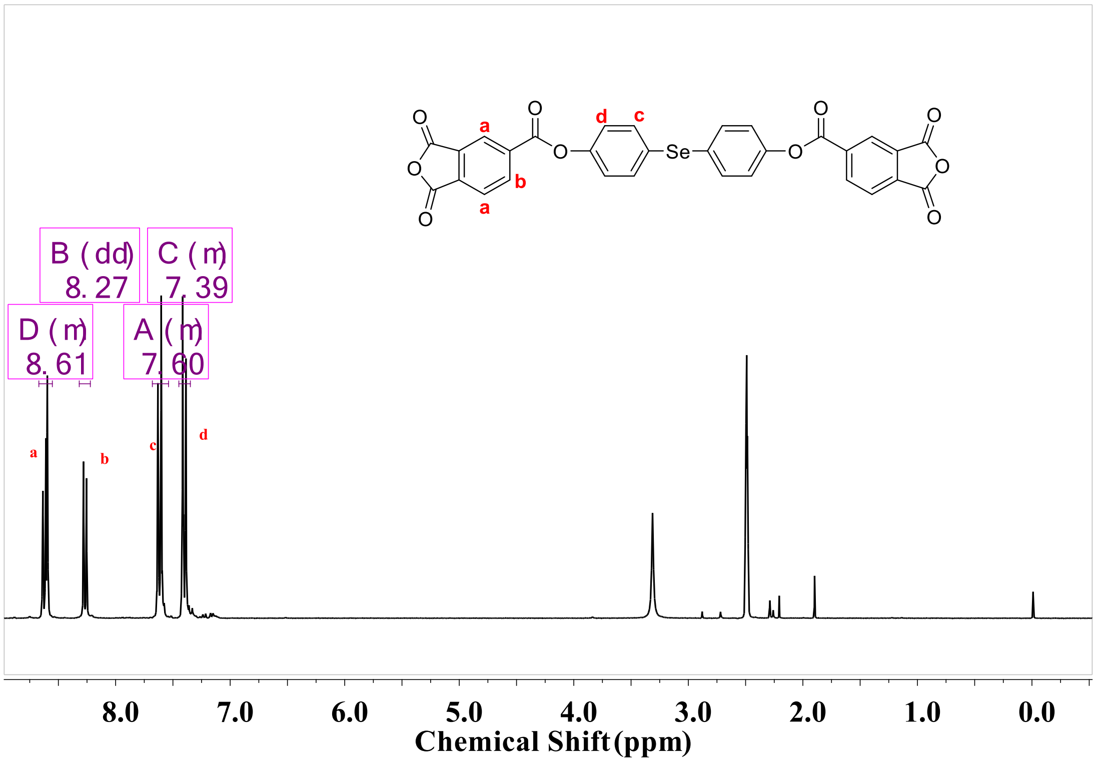

Figure S7. ^{1}H NMR of BDPSD in DMSO-d_6.

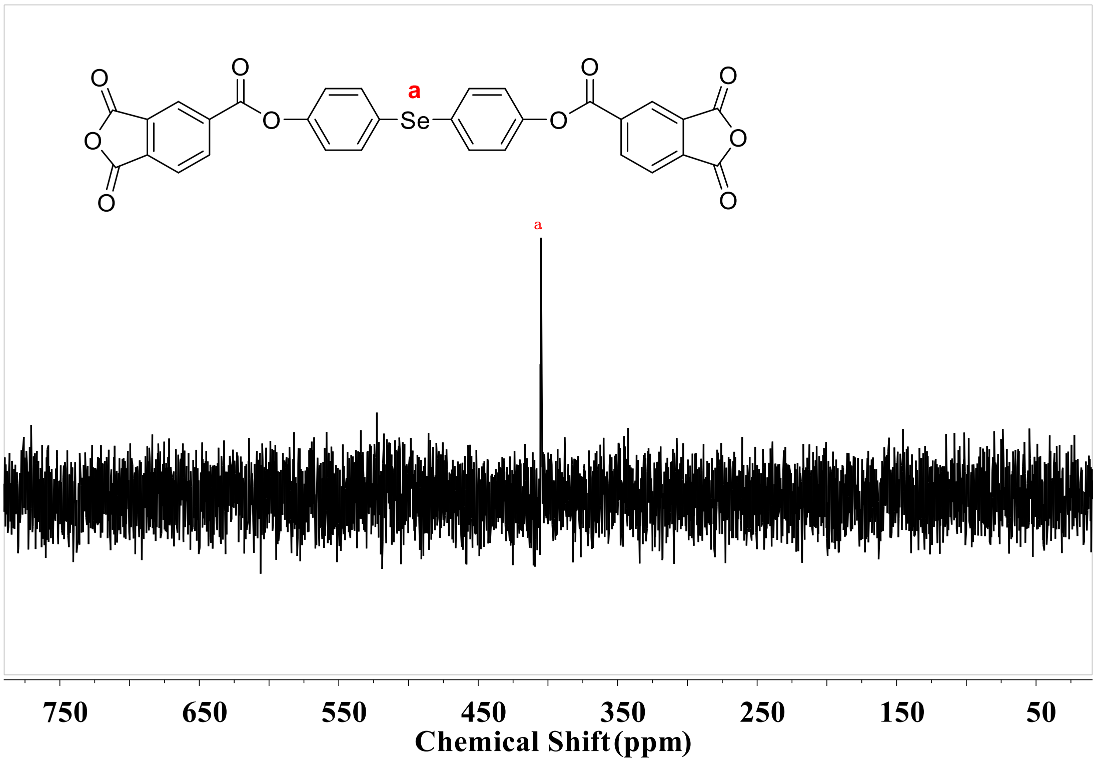

Figure S8. ^{77}Se NMR of BDPSD in DMSO-d_6.

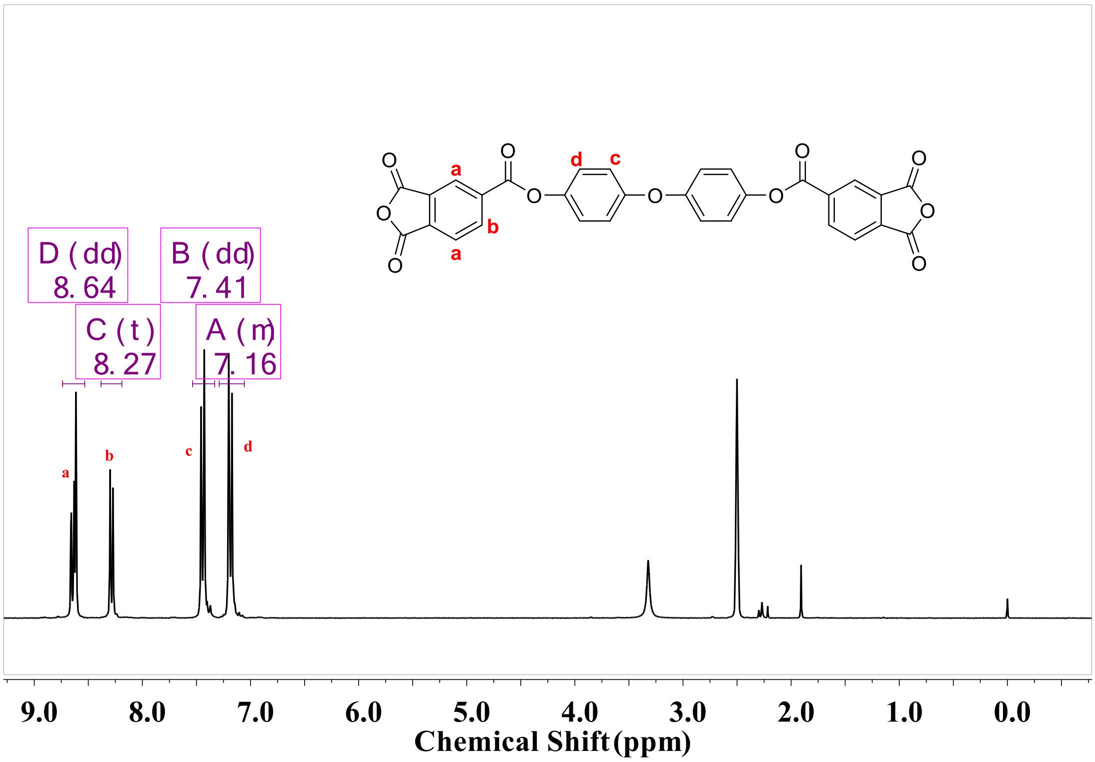

Figure S9. ^{1}H NMR of BDPED in DMSO-d_6.

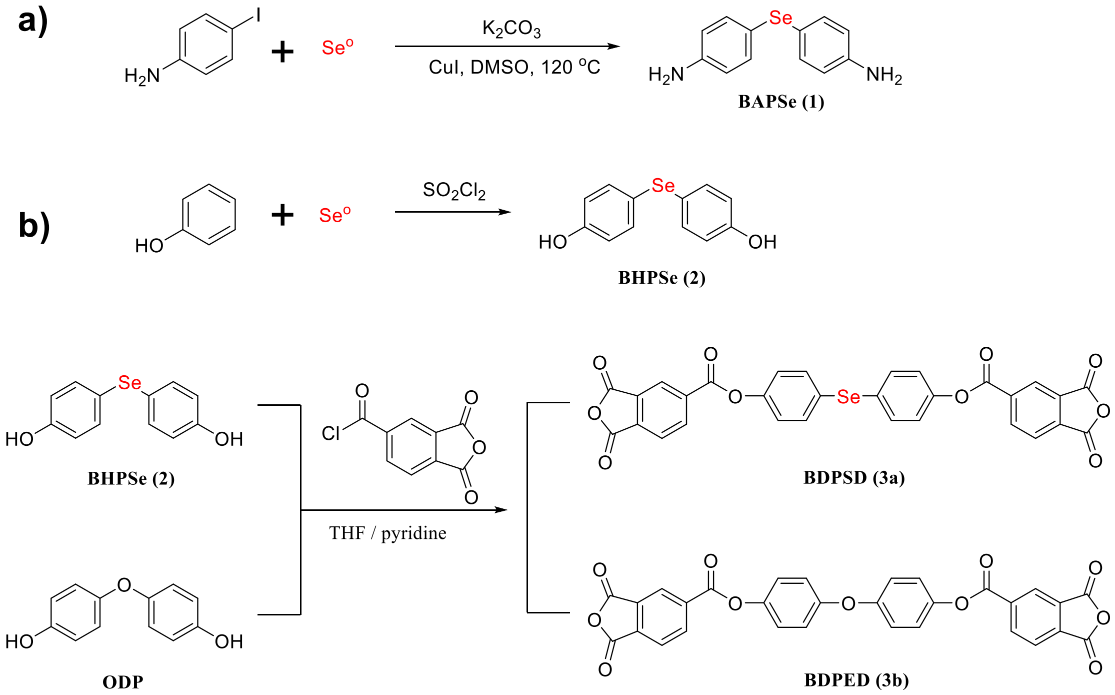

Scheme S1. Synthetic route of monomers (a, synthesis route of BAPS; b, synthesis route of BHPS, BDPSD and BDPED).

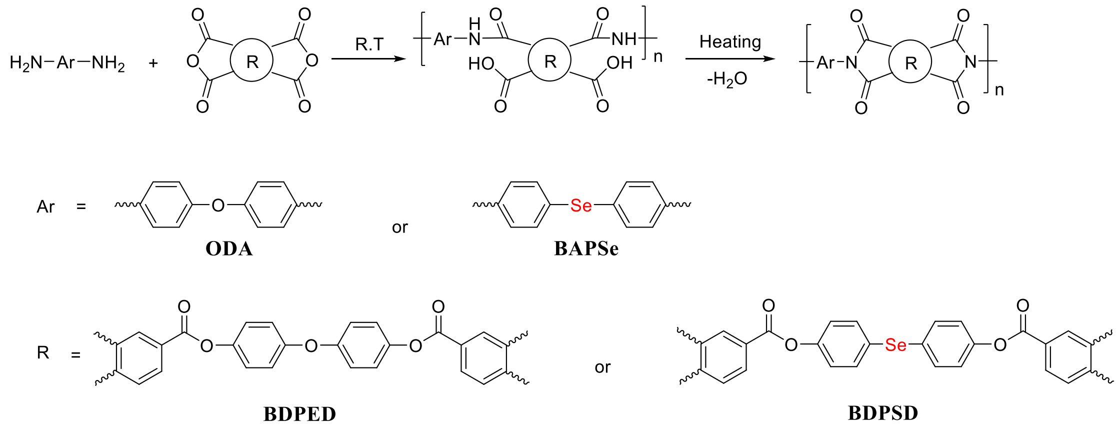

Scheme S2. Preparation procedures and the structures of four PIs with different contents and location of selenium in their repeat units.

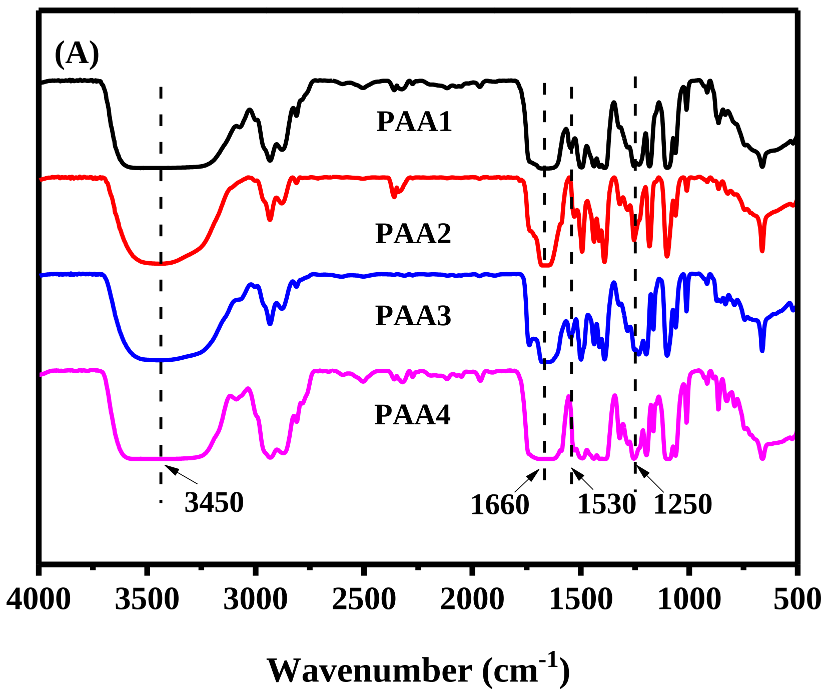

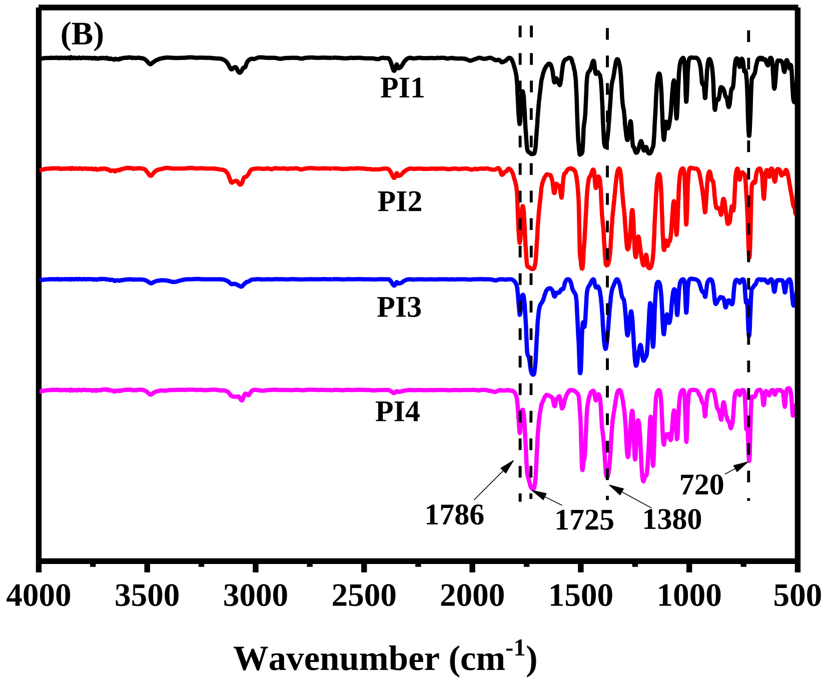

Figure S10. FT-IR spectra of four synthesized PAAs (PAA1-4) (A) and four synthesized PIs (PI1-4) (B) films on the potassium bromide tablet.

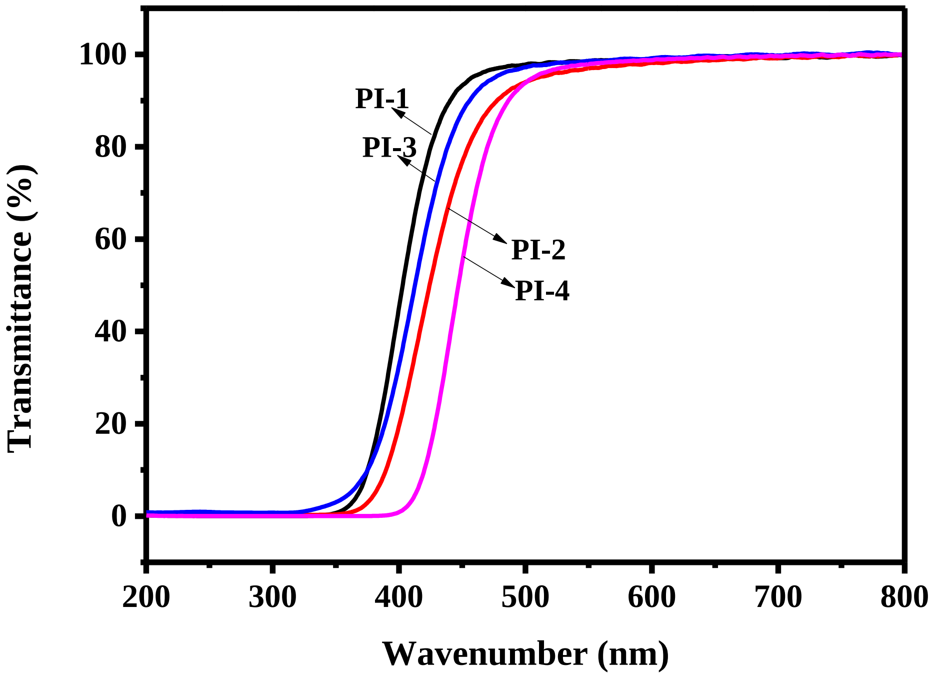

Figure S11. UV-*vis* spectra of four synthesized PIs with about 2 μm thickness.

Table S1. Optical Transmittance, Absorption Edges (λ_E), Calculated Molecular Orbital Energies (ε) of HOMO and LUMO and Energy Band Gaps ($\Delta\varepsilon$) for Monomeric Models of four synthesized PIs.

PIs	T_{400} (%)[a]	T_{200} (%)[b]	λ_E[c] (nm)	ε_{HOMO} (eV)	ε_{LUMO} (eV)	$\Delta\varepsilon$ (eV)
PI-1	45.2	0.2	434	-6.30	-3.11	3.19
PI-2	19.5	0.4	466	-5.91	-3.12	2.79
PI-3	32.6	0.8	451	-6.09	-3.15	2.94
PI-4	0.9	0.1	477	-5.92	-3.15	2.77

[a] Transmittance at 400 nm; [b] Transmittance at 200 nm; [c] the intersection of tangent line of the absorption curve and initial curve.

Table S2. Molecular orbital (MO) diagrams of four synthesized PIs.

PIs	HOMO	LUMO	Length (Å)[a]	Dihedral angle (°)[b]	
				Dianhydride	Diamine
**PI-1**	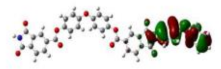	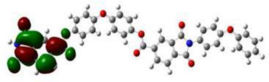	**33.19**	**52.68**	**66.94**
**PI-2**	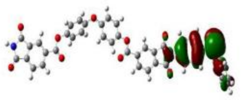	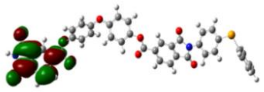	**32.43**	**57.06**	**66.46**
**PI-3**	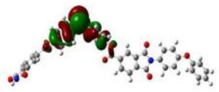	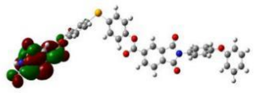	**31.26**	**40.59**	**60.19**
**PI-4**	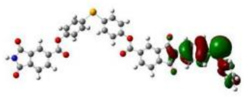	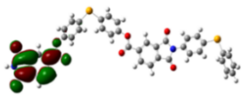	**30.46**	**51.19**	**81.75**

[a] The linear length of one repeat unit in HOMO state.

[b] Dihedral angle between adjacent phenyl group in HOMO of repeat unit.

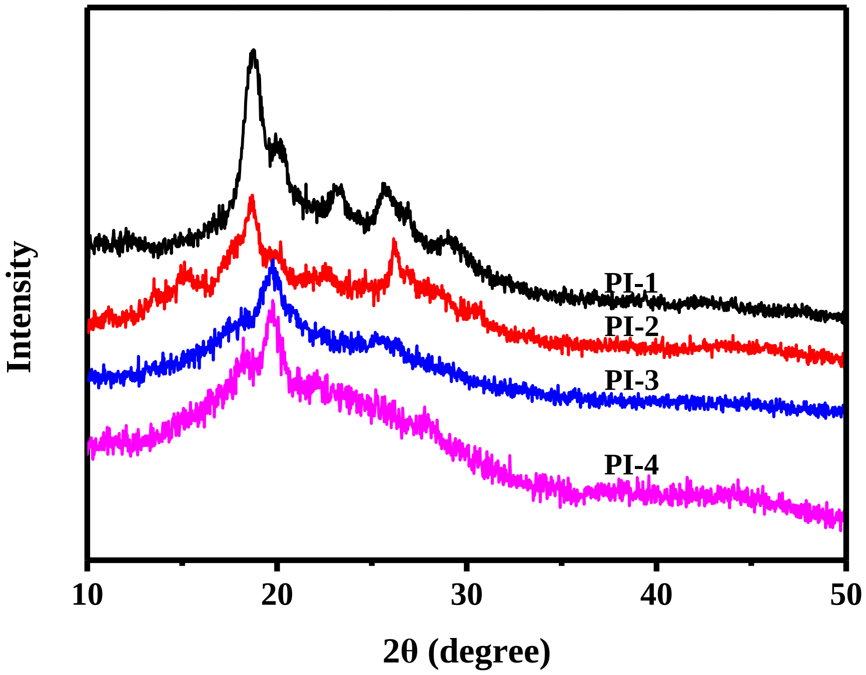

Figure S12. WAXD profiles of four synthesized PIs.

Table S3. Solubility of four synthesized PIs[a].

PIs	DMSO	DMAc	DMF	NMPc	m-Cresol	THF	$CHCl_3$	Acetone
1	-	-	-	-	-	-	-	-
2	-	-	-	-	-	-	-	-
3	-	-	-	-	-	-	-	-
4	-	-	-	-	-	-	-	-

[a] Qualitative solubility measured with 10 mg of the PIs in 1mL of solvent. -, insoluble at room temperature and heating.

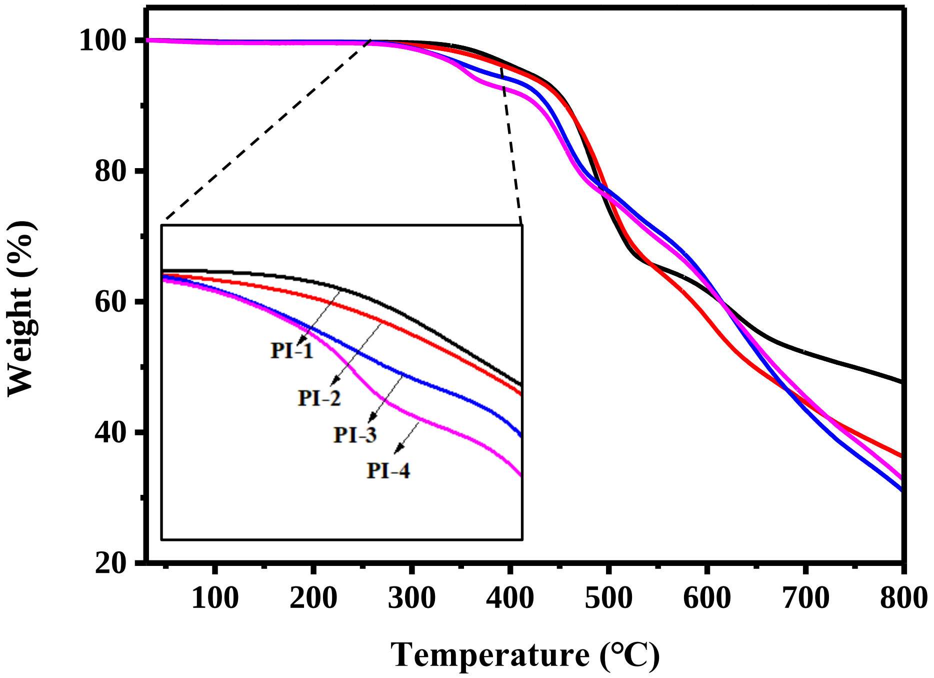

Figure S13. TGA thermographs of four synthesized PIs with N_2 atmosphere.

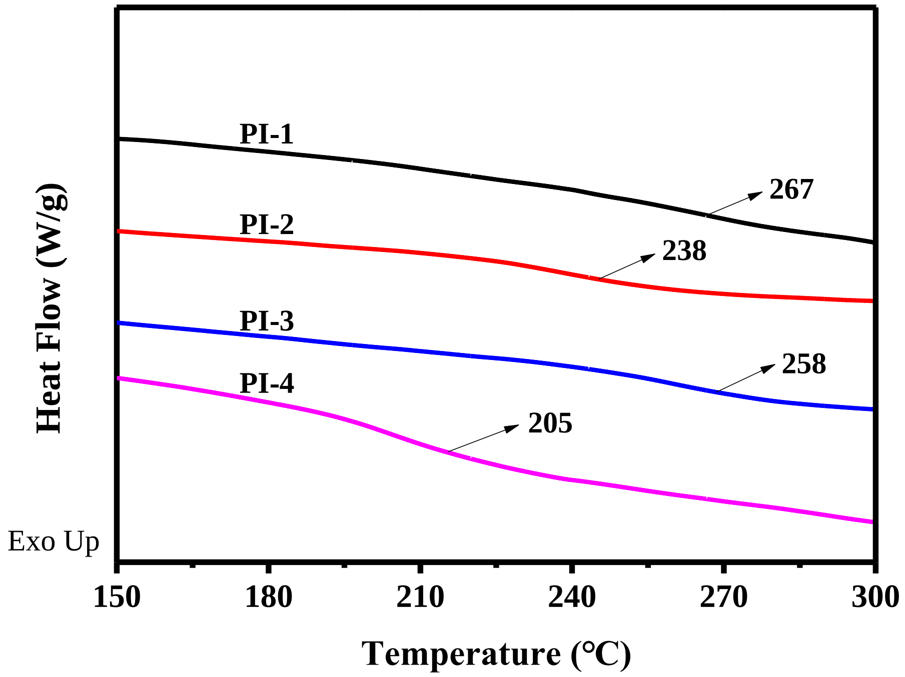

Figure S14. DSC thermographs of four synthesized PIs with N_2 atmosphere. Glass transition temperature of four PIs were labeled.

Table S4. Thermal properties of four synthesized PIs[a]

PIs	T_g (°C)	T_d^5(°C)	T_d^{10}(°C)	Char yield (%)
PI-1	267	417	457	47.5
PI-2	238	411	456	36.2
PI-3	258	376	438	30.8
PI-4	205	356	438	32.5

[a] Tg, glass transition temperature of four PIs; T_d^5, the temperature at 5% weight loss; T_d^{10}, the temperature at 10% weight loss; Char yield, residual weight percentage at 800 °C in N_2 atmosphere.
